# Advancing neurotech justice in youth digital mental health: insights from an interdisciplinary and cross-generational workshop

**DOI:** 10.1038/s44277-025-00052-x

**Published:** 2026-04-08

**Authors:** Craig W. McFarland, Donnella S. Comeau, Sepideh Abdi, Mahsa Alborzi Avanaki, Leo Anthony Celi, Neurotech Justice Workshop Participants, Craig W. McFarland, Sepideh Abdi, Mahsa Alborzi Avanaki, Leo Anthony Celi, Julian Adong, Shaikha Alothman, Manal Brahimi, RuQuan Brown, Cecile Chavane, Donnella S. Comeau, Jack Gallifant, Felix Garcia, Gabriel Làzaro-Muñoz, Eliane Motchoffo, Claire Joy Moss, Derek Ricketts, Paulos Solomon, Takeshi Tohyama, Francis X. Shen, Benjamin C. Silverman, Francis X. Shen, Benjamin C. Silverman

**Affiliations:** 1 University of Oxford, Oxford, UK; 2 Department of Radiology, Beth Israel Deaconess Medical Center, Harvard Medical School Teaching Hospital, Boston, MA USA; 3 Laboratory for Computational Physiology, Massachusetts Institute of Technology; Division of Pulmonary, Critical Care and Sleep Medicine, Beth Israel Deaconess Medical Center; Department of Biostatistics, Harvard T.H. Chan School of Public Health, Boston, MA USA; 4 University of Minnesota Law School & Graduate Program in Neuroscience; Massachusetts General Hospital Center for Law, Brain & Behavior; Harvard Medical School Center for Bioethics; Neurotech Justice Accelerator at Mass General Brigham, a Dana Foundation Center for Neuroscience & Society, Boston, MA USA; 5 Human Research Affairs, Mass General Brigham; Institute for Technology in Psychiatry, McLean Hospital; Harvard Medical School, Boston, MA USA; 6 Mbarara University of Science & Technology, Mbarara, Uganda; 7 Kuwait Healthy Cities, Kuwait City, Kuwait; 8 Bunker Hill Community College, Boston, MA USA; 9 Harvard College, Cambridge, MA USA; 10 Massachusetts Institute of Technology, Cambridge, USA; 11 Department of Radiology, Beth Israel Deaconess Medical Center, Harvard Medical School Teaching Hospital, Boston, USA; 12 Mass General Brigham, Boston, MA USA; 13 Mainstay, Boston, MA USA; 14 Apple, San Francisco, CA USA; 15 Steppingstone, Boston, MA USA; 16 Massachusetts Institute of Technology, Cambridge, MA USA

**Keywords:** Ethics, Policy

## Abstract

Researchers and clinicians are increasingly looking to leverage artificial intelligence (AI) and digital tools to improve psychiatric care. Of particular promise is addressing the youth mental health crisis. Yet, the introduction of AI-enabled digital technologies for psychiatric treatment of young adults raises a host of ethical, legal, and societal issues (ELSI). To provide guidance in addressing these issues, we convened a two-day meeting at the Radcliffe Institute for Advanced Study at Harvard University: *Advancing Neurotech Justice in Mental Health: Insights from an Interdisciplinary and Cross-Generational Workshop*. The meeting brought together a diverse cohort of 17 experts and 5 students from various fields and different countries. In partnership with the MIT Critical Data team, the workshop engaged participants in an interactive Prompt-a-Thon to explore first-hand the potential benefits, biases, and harms related to the use of Large Language Model chatbots in mental health care. This Perspective reports on five principles of digital psychiatry deployment that the workshop participants determined to be the most essential: ensuring accuracy, remaining human-centric, promoting just access, protecting privacy, and providing transparency. We place these five principles within a “Neurotech Justice” framework and discuss how guardrails can be built to promote neurotech justice in digital psychiatry.

## Lay Summary

This article presents findings from an interdisciplinary workshop focused on Neurotech Justice in youth digital mental health, addressing the ethical issues raised by the increasing use of AI-enabled tools to provide mental health services for young adults. Workshop participants, a mix of youth and experts, co-created a framework of five core principles to ensure equitable and ethical deployment: ensuring accuracy, remaining human-centric, promoting just access, protecting privacy, and providing transparency.

There is emerging evidence that young adults in the United States and globally are in the midst of a mental health crisis [1, 2]. This challenge is felt acutely in secondary schools and on college campuses [3]. The rise in mental health needs has created an unprecedented strain on healthcare systems [2, 4]. Modalities that heavily depend on face-to-face consultations are failing to meet the growing need for accessible, affordable, and scalable mental health services. However, new neurotechnology such as digital phenotyping tools offers a potential solution [5]. Technology-based mental health support and treatment platforms are increasingly being developed and deployed to facilitate improved mental health diagnosis and treatment [6, 7], including for college-aged students [8]. Could increasing utilization of neurotechnology be leveraged to deliver improved mental health care for young adults? Maybe, but it is unlikely to be beneficial if the methods used to develop and implement these tools are not accessible, inclusive, and equitable [9]. The accelerating use of AI tools for mental health support has raised pressing concerns that deployment may be outpacing safeguards. For instance, in 2025, two youth suicides were linked to their interactions with large language models for mental health support [10, 11], illustrating the potentially severe consequences of deploying these tools without adequate safeguards.

In this Perspective, we present a framework consisting of five core principles—ensuring accuracy, remaining human-centric, promoting just access, protecting privacy, and providing transparency—to ensure that psychiatry’s implementation of mental health AI tools is done equitably and ethically. We describe these principles as falling within a broader “neurotech justice” framework, which encompasses considerations in the development and deployment of neurotechnologies.

Neurotech justice is a broad concept that can apply to a wide range of neurotechnologies. Neurotechnology covers many applications, including but not limited to deep brain stimulation, brain-computer interfaces, transcranial magnetic stimulation, electroencephalography, and functional magnetic resonance imaging. These technologies have been variously categorized in the literature along dimensions such as invasive vs. non-invasive; clinical vs. research; and read-out vs read-in [12]. In this paper, we focus on one rapidly expanding area in the neurotechnology landscape: digital psychiatry tools such as large language models (LLMs) and related AI-enabled platforms for youth mental health. We focus on LLMs as they are adopted more readily into everyday practice than brain scans or brain implants. LLMs are also being quickly and increasingly utilized in real-world mental health situations, and they can also be directly accessed by nonscientists, as compared to many other neurotechnologies, which require a clinician intermediary. We narrow our focus to digital tools aimed at youth because young adults are among the earliest and most frequent users of AI-enabled mental health tools and may be particularly vulnerable to LLMs’ harms if safeguards are not in place. At the same time, schools and youth-centered institutions provide practical opportunities to operationalize these principles through education, awareness, and participatory design.

**Fig. 1:**
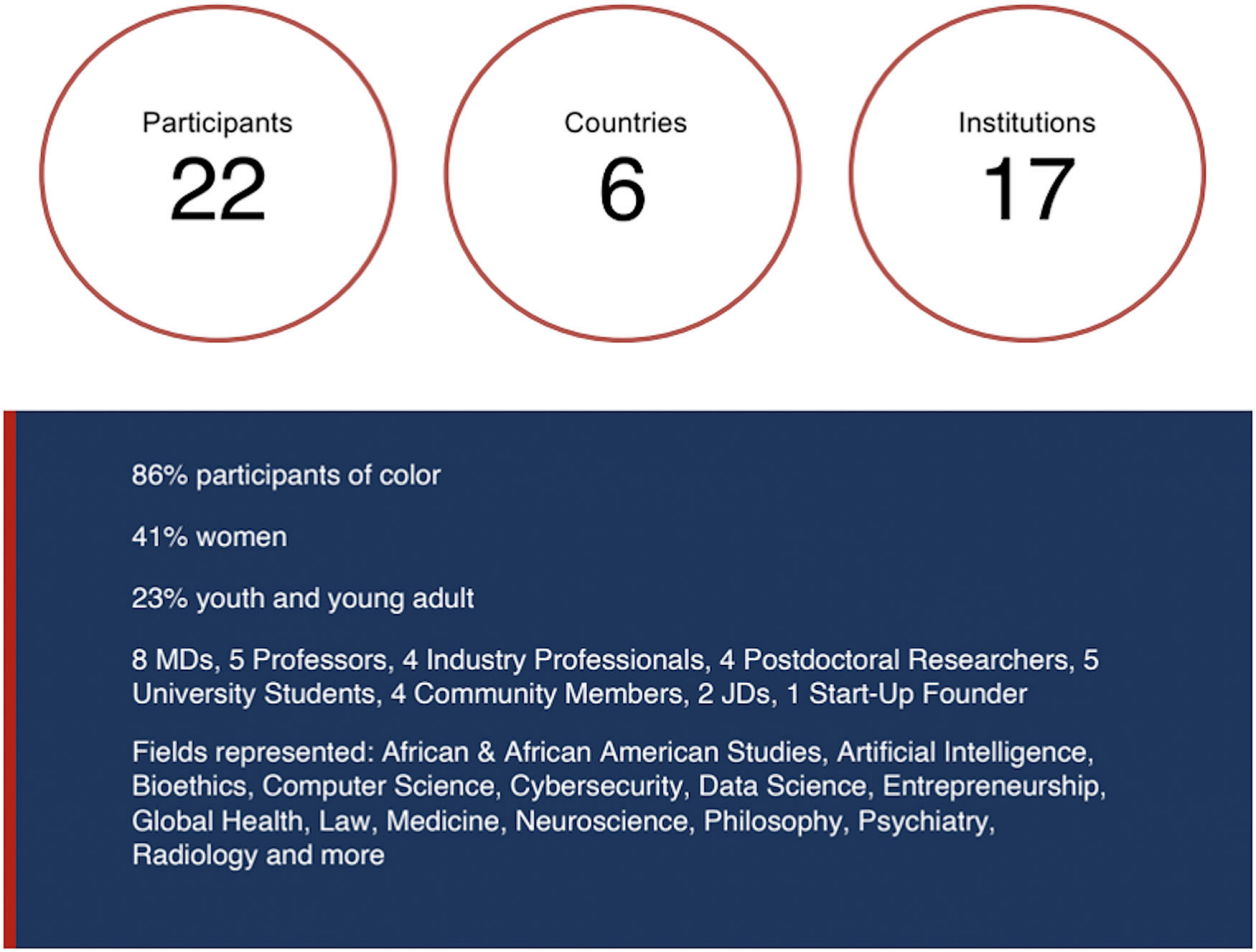
Composition of Neurotech Justice Workshop Participants.

The framework was co-created by an interdisciplinary and multi-generational group, which convened for a two-day workshop at the Radcliffe Institute for Advanced Study at Harvard University: *Neurotech Justice: Engaging Young Adults to Improve Digital Mental Health Tools, Protect Privacy, and Ensure Computational Justice* (May 13-14, 2024). In total, the group convened for a total of 16 h, including five shared meals. The meeting brought together 17 experts from diverse fields and locations (Fig. 1) and five students from different countries to explore the ethical, legal, and societal (ELSI) challenges of integrating neurotechnology and artificial intelligence (AI) into mental health care for young adults. In addition, the workshop featured a presentation by Dr. Kevin Simon, MD, MPH, Boston’s first Chief Behavioral Health Officer, on “Protecting Youth Mental Health,” which provided clinical and public health context for the discussions.

In partnership with the Massachusetts Institute of Technology (MIT) Critical Data team, the workshop engaged participants in an interactive Prompt-a-Thon to explore first-hand the potential benefits, biases, and harms related to the use of LLM chatbots in mental health care. A prompt-a-thon is an interactive exercise in which participants develop different prompts to submit to the LLM and then carefully review the different responses the LLM generates [13]. Rather than simply telling participants that different prompts may lead to different and potentially harmful LLM outputs, a prompt-a-thon approach allows participants to directly engage with AI and observe these effects for themselves. In our workshop, the prompt-a-thon featured prompts related to youth mental health. After participants completed exercises facilitated by the MIT Critical Data team, participants engaged in group discussions about the effects of prompt engineering.

The workshop consisted of a cross-generational cohort composed of physicians and experts, mental health providers, with lived experience of the clinical side of mental health, founders and leaders in tech and AI leaders from various countries, local community members, and college students. This diversity across age, gender, race, ethnicity, geography, training, and expertise was crucial to ensuring that our deliberations included both expert perspectives (e.g., the latest evidence of neurotechnology efficacy) and lived experiences from those directly affected by mental health issues.

We recognize that our framework emerges alongside numerous efforts to develop ethical guidance and regulation of AI. To date, there have been over 200 such guidelines promulgated [14]. These include guidelines from multidisciplinary experts and prominent organizations, international legislation (e.g., the European Union’s ethics guidelines for Trustworthy AI) [15], and state-level regulations in the United States [16].

The novelty of our approach lies not in the final list of principles—which are similar to the core tenets of many of these 200 other guidelines—but rather in the collaborative process that generated them. Generating a framework specific to emerging digital psychiatry tools for youth mental health by test driving those tools with the youth in the room grounded our analysis in the practical realities of those most affected. That our bottom-up approach arrived at a similar outcome as the top-down expert-driven approaches is evidence that our method can be utilized for engaging communities in the process of AI guidance development.

## Addressing the youth mental health crisis with novel neurotechnologies

Since the 2010s, there has been a significant rise in mental health issues among young people, including elementary-aged children [17]. The COVID-19 pandemic further negatively affected youth mental health [18]. Increased rates of loneliness, anxiety, depression, and other mental health disorders have created a surge in demand for mental health services [19]. But a shortage of mental health professionals, long wait times, and limited access to care have left many young people without the support they need [20]. Compared to other age groups, youth with any mental illness are least likely to receive mental health services, and less than half of youth in the US who need mental health care can access it [21].

Innovative digital mental and behavioral health tools have been proposed as part of a multiprong solution to address unmet mental and behavioral needs. Teletherapy platforms and mobile applications have emerged as a vital resource, providing accessible and flexible mental health support in a time where mobile devices are common among youth [22, 23].

Another potentially promising development to improve youth mental health is the use of AI-enabled chatbots and virtual therapists [24]. These tools promise the possibility of real-time support and guidance, offering psychotherapy, healthcare education, training for symptom management, early intervention, and screening for disorders [25]. Moreover, data analytics and machine learning are being harnessed to identify at-risk individuals and tailor interventions to their specific needs [26], improving outcomes and reducing the burden on mental health professionals. Several studies [27, 28] report that young adults are viewing AI-enabled chatbots as a favorable or even preferred mode of psychotherapy, underscoring the potential of AI-enabled chatbots to encourage those who might otherwise avoid seeking help to access mental health support. Together, these emerging neurotechnologies offer promising avenues for youth amidst a growing mental health crisis.

## Risks of deploying AI tools to address youth mental health

While digital mental health tools offer much promise for addressing the youth mental health crisis, it is not yet clear if they will live up to their potential. Participants in our workshop discussed a number of issues that have been identified in the literature, with the group arriving at a focus on three major concerns: accountability for harm, privacy, and equity.

An LLM providing youth mental health advice could offer inaccurate guidance that either discourages professional help, or exacerbates existing distress. Both concerns have been identified by multiple researchers in the field [29, 30]. In addition, LLMs providing youth mental health advice could lead to direct physical harm, such as recommendations for self-mutilation and other high-risk behaviors [31]. In some cases, chatbots have directly recommended homicidal and suicidal action, leading to tragic outcomes among young adults and teens [9, 10, 32]. At present, it is unclear who will be held legally responsible in these types of cases, nor is it clear what regulatory oversight is in place to avoid the replication of such harms [33, 34].

Privacy concerns also emerged as a predominant theme in our discussions. These tools often require the collection of vast amounts of sensitive personal data (e.g., behavioral patterns) to function effectively. Psychiatry, for instance, is beginning to adopt digital phenotyping and AI-driven tools that track participants’ locations, online activity, phone and text message usage, and more [35]. Participants expressed deep concerns that such data could be exploited by third parties. For example, mental health data could be sold to advertisers, insurers, or employers, leading to targeted manipulation, discrimination in hiring or insurance coverage, or stigmatization based on mental health status [36]. The opaque and continuous nature of many data collection practices leaves users, often in a vulnerable state as they seek support for mental health challenges, unaware of what information is being gathered and how it might be used. The potential for data breaches or unauthorized access only heightens privacy concerns, further eroding trust in otherwise promising mental health technologies.

Equity concerns surrounding digital mental health tools include how these technologies may exacerbate existing health disparities rather than alleviate them. A concrete example, illuminated by the prompt-a-thon, was the possibility that an LLM would provide systematically different recommendations to individuals from one socioeconomic or racial group, as compared to another. Participants expressed uneasiness about how such responses could in effect codify bias and perpetuate existing social inequities if not carefully managed, especially as AI systems are trained on data primarily from affluent users. Algorithms trained on biased datasets may inadvertently reinforce discriminatory practices, thereby disproportionately affecting marginalized youth and providing higher rates of substandard care and inaccurate diagnoses for minority users [37].

Relatedly, participants worried about the commodification of mental health care, where a company’s prioritization of profit may contribute to the digital divide and pose financial barriers to underserved communities. Racial and ethnic minorities, for example, are often excluded from accessing digital mental health resources due to systemic barriers like limited internet access, high data costs, and low digital literacy [38]. When financial incentives take precedence, high subscription fees, paywalls, and premium features can create significant cost barriers, preventing vulnerable populations from accessing potentially life-saving mental health support. This profit-driven model directly contradicts the potential of digital mental health tools to expand care in mental health services and instead undermines the promise of these technologies to democratize mental health care. Moreover, limited access among marginalized groups creates a harmful feedback loop of biased data collection and training.

## Co-created principles of neurotech justice

**Fig. 2:**
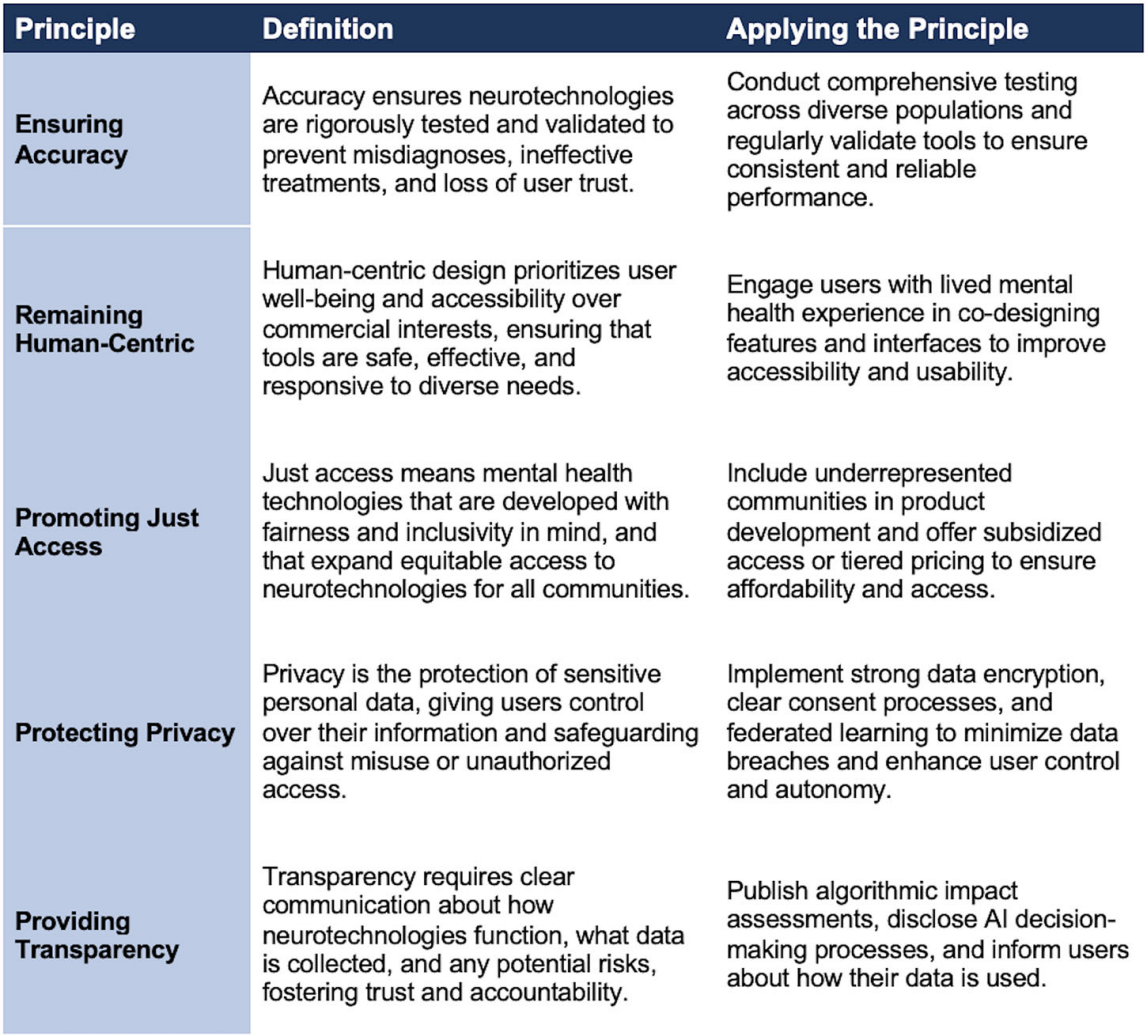
Co-Created Principles of Neurotech Justice.

To help ensure that emerging digital mental health tools are developed and implemented ethically, equitably, and effectively, the workshop participants co-created a set of guiding principles of neurotech justice Fig. 2.

The workshop briefing materials provided relevant background on existing frameworks of neurorights [39]. During the workshop we followed a structured and participatory method to identify principles. Participants first engaged in open discussions to generate a comprehensive list of potential guiding principles and solutions, reflecting diverse perspectives on addressing the challenges in neurotechnology and mental health care. This extensive initial list, totaling over 20 potential key principles, included issues such as cultural sensitivity, harm reduction, and community integration. To refine and prioritize this list, participants provided input through facilitated group discussions and subsequently completed an anonymous survey to rank the proposed principles. Through this iterative approach, the workshop collaboratively distilled the initial ideas into five core principles for advancing neurotech justice in mental health care: (1) ensuring **accuracy**; (2) remaining **human-centric**; (3) promoting **just access** for youth; (4) protecting **privacy**; and (5) providing **transparency**.

### Ensuring accuracy

Accuracy refers to ensuring that the design and deployment of neurotechnologies produce reliable and valid outcomes across populations. Inaccurate tools can lead to misdiagnoses, ineffective treatments, and a loss of user trust. Rushing software and products to market without sufficient validation may result in “technical debt,” where costly harms and inefficiencies arise over time [40]. To improve accuracy, developers must conduct rigorous, representative testing across diverse populations before releasing mental health tools. For example, mental health assessment apps should be tested for diagnostic reliability across different racial, gender, and socioeconomic groups to ensure consistent performance regardless of user identity.

### Remaining human-centric

Human-centric design ensures that the development of neurotechnologies prioritizes the needs, well-being, and experiences of users over the financial interests of developers or commercial stakeholders. This approach emphasizes the design of technologies that are safe, effective, and accessible to all users. A human-centric mental health app could involve participatory design practices, where individuals with lived mental health experiences are directly involved in shaping features, user interfaces, and accessibility options. For instance, integrating customizable settings to accommodate users with disabilities ensures that technology adapts to users rather than forcing users to adapt to the technology.

### Promoting just access

Promoting just access involves integrating metrics around fairness, equity, and inclusivity in neurotechnology development, addressing the systemic barriers and historical harms that have marginalized vulnerable communities in healthcare and technology. This principle seeks to ensure that innovations do not replicate or worsen existing inequities. In practice, this may involve conducting outreach to low-income and underserved populations for participation in pilot programs or ensuring language accessibility in digital health tools. Developers could also implement tiered pricing models or subsidized access to ensure affordability.

### Protecting privacy

Privacy in this context refers to the protection of sensitive personal data collected by digital psychiatry tools. Given that these tools may typically collect highly intimate data, privacy safeguards are critical to maintaining user trust and preventing misuse. To uphold privacy, mental health platforms must implement strong data encryption and clear consent protocols. Where possible, users should have control over their data [41]. For example, employing federated learning allows user data to remain on local devices rather than being stored in centralized servers, reducing the risk of breaches. Additionally, providing transparent data-sharing options enables users to control who accesses their sensitive information.

### Providing transparency

Transparency requires clear and open communication about how digital psychiatry tools function, what data they collect, and the potential risks and limitations of their use. LLMs currently face significant challenges in explaining how their outputs are produced. They are often described as “black boxes” because of their opaque inner workings and lack of interpretability [42]. These limitations mean that LLMs themselves cannot provide trustworthy explanations of their reasoning, and in fact may generate false “explanations” and use deception [43].

One possible solution, being explored in the Explainable AI field, is to utilize a separate “explainer” LLM to interpret the inner computational workings of a “target” LLM [44]. Effective transparency must also go beyond mere disclosure of technical details, ensuring that such information (e.g., algorithmic code) is translated in ways that are understandable and meaningful to users and empowers them to make informed decisions. These considerations are complemented by broader calls for transparency and accountability frameworks to prevent unmonitored or unsafe AI experimentation in healthcare [45]. Potential solutions include model cards and data cards to detail how AI-driven recommendations are generated [46], as well as structured reporting guidelines for research involving LLMs [47]. Additionally, developers should disclose when users are interacting with AI systems versus human professionals and clarify how personal data is used to shape responses.

## Advancing neurotech justice

Pathways to implementing these co-created principles are critical to addressing the concerns posed by novel neurotechnologies and ensuring the potential that these AI-driven mental health tools promise. The workshop concluded with a collaborative exercise in which participants identified actionable next steps for advancing neurotech justice in mental health care. This process generated concrete strategies focused on empowering youth, engaging policymakers, and fostering cross-sector collaboration to ensure the ethical development and deployment of neurotechnologies.

Immediate next steps included amplifying youth voices and actively involving young adults in shaping the future of neurotechnology. As early adopters of digital technologies and a population increasingly targeted by mental health interventions, young people offer valuable perspectives on how these tools can be designed to be more accessible, effective, and responsive to user needs. Involving youth through advisory boards, participatory design initiatives, and youth-led advocacy ensures that their experiences and insights inform the development of ethical and equitable mental health technologies.

Long-term efforts must address the historical systemic racial, gender, and economic inequities embedded in the current healthcare systems. This requires proactive measures to ensure that neurotechnologies meaningfully prioritize inclusivity and equitable access. Efforts should include investing in community-engaged research, ensuring diverse representation in product development and clinical trials, and designing technologies that are culturally responsive and accessible to marginalized populations. Outreach to colleges and universities is essential, but meaningful engagement must also extend to secondary and elementary schools. Such outreach should be led collaboratively by mental health professionals, academic societies, physicians across specialties—including pediatricians—educators, and academic leaders. Doing so will best support age-appropriate education about AI-enabled large language models and their mental health implications across all levels of community engagement. It is equally important to examine who is involved in developing, training, and deploying these models, as the perspectives and power dynamics of those shaping the systems influence both model behavior and downstream harms. Additionally, funding mental health initiatives in underserved communities, expanding access to the internet and other digital infrastructures, and supporting programs that build digital literacy are critical to bridging existing gaps.

To ensure these efforts have a sustained impact, systemic change must be driven by both industry leadership and supportive policy frameworks. Engaging corporate leaders, particularly C-suite executives at technology companies, will be critical to the integration of ethical principles into product development, corporate strategy, and regulatory frameworks. Collaborating with policymakers is key to enforcing legal protections and regulations that safeguard the advances of neurotechnology.

Further interdisciplinary collaborations, research, and ongoing advocacy efforts are essential to raise awareness about neurotech justice. This involves increasing literacy around neurotechnology among the public and educating policymakers, healthcare professionals, and technology developers about the ethical considerations and potential impacts of neurotechnology. Such efforts must also build a rigorous evidence base that directly addresses the methodological shortcomings that limited earlier research on digital harms, ensuring that studies at the intersection of AI and youth mental health generate reliable and actionable knowledge, especially for the public. Strengthening public understanding and engagement not only empowers individuals to make informed decisions but also holds developers accountable for responsible innovation. Taking these steps will enable digital psychiatry tools to fulfill their potential and contribute to the democratization and improvement of mental health care for all.

### Citation diversity statement

The authors have attested that they made efforts to be mindful of diversity in selecting the citations used in this article.
